# Relationship of work-family conflict, self-reported social support and job satisfaction to burnout syndrome among medical workers in southwest China: A cross-sectional study

**DOI:** 10.1371/journal.pone.0171679

**Published:** 2017-02-16

**Authors:** Shujuan Yang, Danping Liu, Hongbo Liu, Juying Zhang, Zhanqi Duan

**Affiliations:** 1 Department of Health Related Social and Behavioral Science, West China School of Public Health, Sichuan University, Chengdu, Sichuan Province, China; 2 Department of Health Statistics, School of Public Health, Chinese Medical University, Shenyang, Liaoning Province, China; 3 Department of Epidemiology and Health Statistics, West China School of Public Health, Sichuan University, Chengdu, Sichuan Province, China; 4 Center for Health Information of Sichuan Province, Chengdu, Sichuan Province, China; University of Rochester, UNITED STATES

## Abstract

**Background:**

Burnout is a psychosomatic syndrome widely observed in Chinese medical workers due to the increasing cost of medical treatment, excessive workload, and excessive prescribing behavior. No studies have evaluated the interrelationship among occupational burnout, work-family conflict, social support, and job satisfaction in medical workers. The aim of this study was to evaluate these relationships among medical workers in southwest China.

**Methods:**

This cross-sectional study was conducted between March 2013 and December 2013, and was based on the fifth National Health Service Survey (NHSS). A total of 1382 medical workers were enrolled in the study. Pearson correlation analysis and general linear model univariate analysis were used to evaluate the relationship of work-family conflict, self-reported social support, and job satisfaction with burnout syndrome in medical workers.

**Results:**

We observed that five dimensions of job satisfaction and self-reported social support were negatively associated with burnout syndrome, whereas three dimensions of work-family conflict showed a positive correlation. In a four-stage general linear model analysis, we found that demographic factors accounted for 5.4% of individual variance in burnout syndrome (F = 4.720, P<0.001, R^2^ = 0.054), and that work-family conflict, self-reported social support, and job satisfaction accounted for 2.6% (F = 5.93, P<0.001, R^2^ = 0.080), 5.7% (F = 9.532, P<0.001, R^2^ = 0.137) and 17.8% (F = 21.608, P<0.001, R^2^ = 0.315) of the variance, respectively. In the fourth stage of analysis, female gender and a lower technical title correlated to a higher level of burnout syndrome, and medical workers without administrative duties had more serious burnout syndrome than those with administrative duties.

**Conclusions:**

In conclusion, the present study suggests that work-family conflict and self-reported social support slightly affect the level of burnout syndrome, and that job satisfaction is a much stronger influence on burnout syndrome in medical workers of southwest China.

## Introduction

Burnout is a psychosomatic syndrome with three dimensions: emotional exhaustion, feelings of depersonalization, and reduced personal accomplishment [1]. With improvements in the social security system and reform of the medical system in China, the accompanying increasing cost of medical treatment, excessive workload, excessive prescribing behavior, tense doctor-patient relationships, burnout syndrome is common in medical workers [2–4]. A recent national survey of the fourth national health services indicated that job satisfaction influences medical workers' burnout and intention to leave their organization [5, 6]. The recent surge in medical disputes, such as violent riots, attacks, and protests in hospitals, have greatly increased the psychological burden for medical workers [4]. Tasks related to scientific research also add to the pressure on medical workers. If these pressures cannot be relieved, emotional exhaustion would result in burnout.

Work-family conflict is a phenomenon first proposed by Greenhaus et al. (1985). They reported that changes in the work environment (promotion and further education) would occupy more family time, while marriage and births of children could influence work efficiency [7]. Frone and Michael et al. found that good family support reduces individuals' negative experiences at work [8], and other studies confirm that the work-family relationship is associated with burnout syndrome [9–11].

Lazarus and Folkman proposed the transaction model of stress, and indicated that perceived social support is based on an individual's assessment of the support available in a given situation, including availability of support and satisfaction with support [10]. Cohen and Wills report that having a source of support available in a stressful situation can significantly decrease its harmful impact [12]. By seeking social support, individuals can find sympathy or help from others and obtain effective coping strategies; conversely, individuals would experience burnout syndrome if they do not get social support [13].

Job satisfaction is an important variable for predicting burnout syndrome [14–16] that affects burnout syndrome through organizational commitment [17–20]. Moreover, intrinsic and extrinsic job satisfaction significantly influences burnout in clinical physicians and nurses through social support, work environment, and work hours [21, 22]. Thus, job satisfaction is a meaningful factor in occupational burnout syndrome. Previous studies in various countries have investigated the correlation of job satisfaction with burnout among corporate employees and nurses [23–28], but few have reported on these correlations in Chinese physicians and public health workers [6].

The concept of burnout was first proposed by Freudenburger in the 1970s, and is widely discussed in clinical psychology [29]. Burnout means the wearing out of an individual due to excessive demands on his or her resources. According to Schaufeli’s theory, burnout may cause exhaustion, result in indifference toward work, and decrease job satisfaction and professional efficacy [30]. The correlation between burnout and job satisfaction has been examined in many previous studies [24, 31, 32]. Some have reported that lack of social support at work significantly contributes to occupational burnout [33, 34]. Although many studies have investigated the relationship among job satisfaction, social support, and occupational burnout, none has evaluated the interrelationship of occupational burnout, work-family conflict, social support, and job satisfaction among physicians, nurses, and public health workers. Therefore, we carried out a cross-sectional study to estimate the role of work-family conflict, self-reported social support, and job satisfaction in burnout syndrome among medical workers in southwest China.

## Materials and methods

### Ethics

Our investigation was based on the fifth National Health Service Survey (NHSS), which was designed by an expert panel from the Center for Health Statistics and Information of the Ministry of Health of China. The fifth NHSS in Sichuan Province was approved by the ethics committee of the Center for Health Statistics and Information of the Ministry of Health of China. Human rights and ethics issues were taken into consideration when the survey was designed. All included subjects voluntary participated in our study and signed informed consents before enrollment, and their information was kept completely anonymous.

### Participants and sampling

The NHSS in Sichuan province was conducted between March 2013 and December 2013. All medical workers who had a practicing qualified certificate on file in the third-class comprehensive hospitals, second-class hospitals, community health service centers, and township hospitals of Sichuan Province were eligible to be study subjects. Multistage stratified random sampling was used to acquire the study sample. In the first stage, 14 cities were randomly selected from among 21 prefecture-level cities. In the second stage, all the third-class comprehensive hospitals and some of the second-class hospitals were selected in the 14 cities, and a total of 70 towns and communities were randomly selected in these cities. All the community health service centers or township hospitals in the 70 towns or streets were enrolled in the investigated medical institutions. In the third stage, a total of 20 clinical physicians and 10 nurses were selected from each third-class comprehensive and second-class hospital by a simple random sampling method. Five physicians, three nurses, and two public health workers were randomly selected from each community health service center and township hospital.

### Measuring instruments

To gather data we used a six-page questionnaire, which included five parts on demographic variables, occupational burnout, work-family conflict, self-reported social support, and job satisfaction (S1 and S2 Tables). The questionnaire was accompanied by an introduction to the purpose of the study, and was designed by the National Health and Family Planning Commission of the People's Republic of China. Our previous study and other studies have used this questionnaire to evaluate job satisfaction in medical workers [35, 36]. Its validity and reliability have been verified in a previous study [36].

Part one of the questionnaire describes basic socio-demographic characteristics, including age, sex, marital status, professional categories, educational background, technical title, years of service, administrative duties, and grade of medical institution.

Part two pertains to indicators of burnout syndrome, including emotional exhaustion, job dedication, and job engagement. Emotional exhaustion is estimated by six items, job dedication is estimated by five items, and job engagement is evaluated by six items. The total score for each dimension is the sum of scores of all items. Each item is expressed with a seven-point Likert scale from 0 (never) to 6 (always). For each dimension, a higher score indicates a higher level of burnout syndrome.

Part three investigates work-family conflict and is estimated with three dimensions: conflicts of time, conflicts of behavior, and conflicts of pressure. Conflicts of time is assessed by three items, conflicts of behavior is evaluated by five items, and conflicts of pressure is estimated by one item. The total score of each dimension is the sum of scores of all items. Each item is expressed by a five-point Likert scale from 1 (strongly disagree) to 5 (strongly agree). A higher score indicates higher conflict between work and family.

Part four describes self-reported social support and is estimated by three items. Each item consists of a five-point Likert scale from 1 (strongly disrespectful/very bad) to 5 (strongly respectful/very good). The total score of self-reported social support is the sum of scores for all items. A higher score indicates a higher level of self-reported social support.

Part five assesses job satisfaction, including satisfaction with the job itself, the work environment, job rewards, organizational management, and the medical practice environment. Satisfaction with the job itself, the work environment, and job rewards are each estimated by two items. Satisfaction with organizational management and the medical practice environment are each estimated by one item. Each item includes a six-point Likert scale from 1 (strongly disagree) to 6 (strongly agree). The total score of each dimension is the sum of scores for all items. For each dimension, a higher score indicates a higher level of job satisfaction.

### Data collection

A cross-sectional study design was used in our study, which was conducted between March 2013 and December 2013. The investigation was completed by trained investigators who received uniform training. All participants were informed the general purpose of our study, but not the research hypothesis, before agreeing to participate. The questionnaire was self-administered. Questionnaires were excluded from analysis if they had incomplete data on socio-demographic characteristics, or three or more items missing of any part of the questionnaire.

### Statistical analysis

Continuous variables are expressed as means ± standard deviations (SD), and categorical variables are displayed as percentages and frequencies (%). The correlation of burnout syndrome with work-family conflict, self-reported social support, and job satisfaction was analyzed by Pearson correlation analysis, and the results were expressed by the Pearson correlation coefficient. A general linear model univariate analysis was used to estimate the effects of demographic factors, work-family conflict, self-reported social support, and job satisfaction on the level of burnout syndrome, and burnout syndrome was treated as a dependent variable. We also evaluated changes in significance and explained variance in independent variables by building general linear models.

Using general linear model univariate analysis, we introduced independent variables one by one in four models. In the first model, we only used demographic factors as independent variables. In the second model, we added work-family conflict into the general linear regression model based on Model 1. In the third model, we added self-reported social support as an independent variable to Model 2. In the fourth model, we added job satisfaction into an analysis based on Model 3. We determined changes in the significance, parameter size, and variance of burnout syndrome caused by each of the independent variables. The variance in burnout syndrome caused by each of the independent variables was estimated by R^2^. Statistical analyses were carried out by the software IBM SPSS Statistics for Windows, Version 20.0. (IBM Corp., Armonk, NY, USA). P < 0.05 was considered to indicate statistically significant difference.

## Results

A total of 1382 medical workers were enrolled in the sample group. Of these, 807 were clinical physicians, 404 were nurses, and 171 were public health workers. The socio-demographic characteristics of investigated subjects are shown in Table 1. Of the 1382 medical workers, 59.6% were female, 71.2% were above 30 years of age, 74.7% had attained a bachelor degree or above, 40.0% had fewer than 10 service years, and 77.0% had no administrative duties.

**Table 1:** Socio-demographic characteristics of investigated subjects.

	Characteristics	Subjects (n = 1382)	Proportion (%)
Age (years)	≤29	398	28.799
	30–39	445	32.200
	40–49	365	26.411
	≥50	174	12.590
Sex	Female	823	59.551
	Male	559	40.449
Marital status	Single	262	18.958
	Married	1078	78.003
	Divorced	42	3.039
Professional categories	Clinicians	807	58.394
	Nurses	404	29.233
	Public health physicians	171	12.373
Education background	Junior college or below	349	25.253
	Bachelor	614	44.428
	Master	362	26.194
	Doctor or above	57	4.124
Technical title	No title	114	8.249
	Medical assistant	235	17.004
	Resident physician	517	37.410
	Attending physician	371	26.845
	Above associate chief physician	145	10.492
Service years (years)	≤9	553	40.014
	10–19	367	26.556
	20–29	274	19.826
	≥30	188	13.603
Administrative duties	No	1064	76.990
	Yes	318	23.010
Grade of medical institutions	Third-class	625	45.224
	Second-class	534	38.640
	Community health service centers and township hospitals	223	16.136

The mean scores for emotional exhaustion, job dedication, and job engagement were 14.778±7.857, 14.249±8.148, and 10.513±6.697, respectively (Table 2). The level of work-family conflict was high, with mean scores for conflicts of time, behavior, and pressure of 11.339±3.194, 10.133±3.131, and 10.873±3.354, respectively. The mean score of self-reported social support was 9.574±2.329. Levels of job satisfaction were relatively high, with mean scores for satisfaction with the job itself, work environment, job rewards, organizational management, and medical practice environment of 8.878±2.264, 8.659±1.992, 7.215±2.545, 4.773±1.208, and 3.578±1.370, respectively.

**Table 2:** Mean values of burnout syndrome, work family conflict, social support at work and job satisfaction of the investigated subjects.

Variables		Mean value (mean±SD)
Burnout syndrome	Emotional exhaustion	14.778±7.857
	Job dedication	14.249±8.148
	Job engagement	10.513±6.697
Work family conflict	Conflict of time	11.339±3.194
	Conflict of behavior	10.133±3.131
	Conflict of pressure	10.873±3.354
Self-reported social support		9.574±2.329
Job satisfaction	Job-itself satisfaction	8.878±2.264
	Work environment satisfaction	8.659±1.992
	Job rewards satisfaction	7.215±2.545
	Organizational management satisfaction	4.773±1.208
	Medical practicing environment satisfaction	3.578±1.370

The correlation of burnout syndrome with job satisfaction, work-family conflict, and self-reported social support was evaluated by Pearson correlation analysis (Table 3). Burnout syndrome was positively related to three dimensions of work-family conflict (P<0.001), but negatively correlated with five dimensions of job satisfaction (all P values<0.001). Burnout syndrome was negatively associated with self-reported social support (P<0.001).

**Table 3:** Pearson correlation analysis of the relationship of work-family conflict, social support at work, and job satisfaction with burnout syndrome.

Variable	Item measured	Pearson correlation coefficient with burnout syndrome	P value
Work-family conflict	Conflict of time	0.063	0.020
	Conflict of behavior	0.111	<0.001
	Conflict of pressure	0.155	<0.001
Self-reported social support		-0.286	<0.001
Job satisfaction	Job-itself satisfaction	-0.479	<0.001
	Work environment satisfaction	-0.313	<0.001
	Job rewards satisfaction	-0.362	<0.001
	Organizational management satisfaction	-0.284	<0.001
	Medical practice environment satisfaction	-0.242	<0.001

The influence of demographic factors, work-family conflict, self-reported social support, and job satisfaction on burnout syndrome was analyzed by a four-stage general linear model (Table 4). In the general linear regression models, work-family conflict, self-reported social support, and job satisfaction were considered as principal predictors.

**Table 4:** General linear model analysis with burnout syndrome dimensions as a dependent variable.

Variable		Model 1	P value	Model 2	P value	Model 3	P value	Model 4	P value
		F value		F value		F value		F value	
Social-demographic factors	Age	0.079	0.971	0.286	0.835	0.654	0.580	0.364	0.779
	Sex	2.381	0.123	3.209	0.073	4.981	0.026	5.977	0.015
	Marital status	0.213	0.808	0.419	0.658	0.134	0.875	0.375	0.687
	Educational background	0.621	0.601	0.709	0.547	0.338	0.798	0.222	0.881
	Professional category	1.174	0.309	0.787	0.456	0.865	0.421	0.697	0.498
	Technical title	1.945	0.101	2.293	0.058	2.880	0.022	5.024	0.001
	Years of service	1.599	0.188	2.074	0.102	2.377	0.068	1.776	0.150
	Administrative duties	10.190	0.001	8.278	0.004	7.938	0.005	4.076	0.044
	Grade of medical institution	0.623	0.537	0.514	0.598	0.120	0.887	0.335	0.716
Work-family conflict	Conflict of time			4.895	0.027	7.790	0.005	6.818	0.009
	Conflict of behavior			4.329	0.038	1.452	0.228	0.143	0.706
	Conflict of pressure			17.418	<0.001	7.721	0.006	0.681	0.409
Self-reported social support						88.343	<0.001	19.931	<0.001
Job satisfaction	Job-itself satisfaction							117.237	<0.001
	Work environment satisfaction							2.989	0.084
	Job rewards satisfaction							4.391	0.036
	Organizational management satisfaction							9.550	0.002
	Medical practice environment satisfaction							1.447	0.229
Correlation model		4.720	<0.001	5.93	<0.001	9.532	<0.001	21.608	<0.001
Adjusted R^2^			0.054		0.080		0.137		0.315

In Model 1, our analysis indicated that demographic factors contributed a 5.4% criterion variance in burnout syndrome (F = 4.720, P<0.001, R^2^ = 0.054). In Model 2, we added a work-family conflict dimension into the analysis based on Model 1, and observed that it accounted for a 2.6% variance in burnout syndrome (F = 5.93, P<0.001, R^2^ = 0.080). In Model 3, we added self-reported social support to Model 2, and found that it explained 5.7% of the variance in burnout syndrome (F = 9.532, P<0.001, R^2^ = 0.137). In Model 4, job satisfaction dimensions were brought into the analysis based on Model 3, and accounted for 17.8% of the variance in burnout syndrome (F = 21.608, P<0.001, R^2^ = 0.315).

In Model 4, female gender emerged as a predictor of burnout syndrome compared to male [*b* = -2.937, 95% CI = (-5.294, -0.580), F = 5.977, P = 0.015]; a lower technical title was related to higher level of burnout syndrome [*b* = 10.727 for no title, 95% CI = (5.026, 16.429), P<0.001; *b* = 11.282 for medical assistant, 95% CI = (60184, 16.381), P<0.001; *b* = 8.228 for resident physician, 95% CI = (4.131, 12.326), P<0.001; b = 5.687 for attending physician, 95% CI = (1.925, 9.450), P = 0.003; F = 5.024, P = 0.001; reference group: above associate chief physician]; and lack of administrative duties was associated with higher level of burnout syndrome [*b* = 2.618, 95% CI = (0.074, 5.162), F = 4.076, P = 0.044] relative to workers with administrative duties. Conflict of time between work and family was correlated with burnout syndrome [*b* = -0.067, 95% CI = (-0.118, -0.017), F = 6.818, P = 0.009]. Self-reported social support [*b* = -0.130, 95% CI = (-0.187, -0.073), F = 19.931, P<0.001], job-itself satisfaction [*b* = -0.331, 95% CI = (-0.392, -0.271), F = 117.237, P<0.001], job rewards satisfaction [*b* = -0.058, 95% CI = (-0.113, -0.004), F = 4.391, P = 0.036] and organizational management satisfaction [*b* = -0.076, 95% CI = (-0.124, -0.028), F = 9.550, P = 0.002] were negatively associated with level of burnout syndrome.

## Discussion

This study investigated the relationship of social-demographic factors, work-family conflict, self-reported social support, and job satisfaction with burnout syndrome among medical workers in southwestern China. We observed that female gender, having a lower technical title and lacking administrative duties were positive predictors for burnout syndrome. Moreover, we found that job satisfaction greatly influenced the level of burnout syndrome, and that work-family conflict and self-reported social support had smaller effects.

In our general linear model analysis, we observed that 2.6% of the variance in burnout syndrome was explained by work-family conflict, principally conflicts of time. A recent meta-analysis of 20 published studies indicated that nursing professionals are vulnerable to depression when they experience conflicts between family and work [37], and depression may promote burnout syndrome. Shanafelt et al. performed a large sample study of 24,922 American surgeons, and reported that work-family conflict was independently associated with burnout [9]. Only 36% of participating surgeons felt that they had enough time for their personal and family life [9]. Martini performed a study in 307 medical workers, and reported that strong work-family conflicts significantly contributed to burnout syndrome [11]. Our findings are in line with such previous results. Time spent taking care of children and housework can impact energy devoted to work [8, 11], while family support helps equip individuals with more energy, confidence, and enthusiasm for their work.

Self-reported social support explained 5.7% of the variance in burnout syndrome in our study. Social support could bring more confidence, satisfaction, and enthusiasm in work, and high social support equates to great social status [10]. Rzeszukek M et al. indicate that the level of burnout symptoms is related to perceived social support [38]. Richa et al. performed a study in French workers, and reported that lack of social support from supervisors, colleagues, and the social community at work, along with work-family conflict, significantly increased burnout [39]. These studies indicate that strong social support at work could reduce burnout syndrome, which supports the findings of our study.

We found that job satisfaction accounted for 17.8% of the variance of burnout syndrome, making it a great influence on the level of burnout syndrome compared with other factors. Satisfaction with the job itself, job rewards, and organizational management each had significant negative associations with burnout. High satisfaction with job rewards corresponds with high income, which would promote individuals' enthusiasm for work and reduce their negative feelings about it [39]. Organizational management includes system construction and leadership behavior; satisfaction with organizational management indicates high-quality leadership and support from supervisors. Zhang et al. reported that satisfaction with the job itself, job rewards, and organizational management were negatively correlated with emotional exhaustion, which directly affects burnout syndrome [6]. However, satisfaction with the work environment and environment for medical practice did not influence burnout [6]. These results are in line with our findings.

Females had a higher level of burnout syndrome in our general linear model analysis than males. Similarly, one study of 5558 neurologists reported that females had lower levels of job satisfaction than males [23]. In general, females are not only responsible for paid work, but also spend more time on housework and caring for children than do males. In addition, a recent study indicates that female physicians more often work in part-time jobs than males, and leading positions are mostly held by male physicians [40]. Therefore, female physicians are more vulnerable to high levels of emotional exhaustion and depression compared to males [40]. These factors may result in burnout syndrome at work. Female medical workers should be given more care and support in their work.

We observed that the medical workers with lower-level technical titles had more burnout syndrome than those with higher-level titles. A recent study similarly indicated that different technical titles were associated with different levels of burnout syndrome [41]. Medical workers with lower technical titles tend to have lower income, higher pressure to attain promotions, fewer social resources, and more energy devoted to coping with problems in work, which may influence their job satisfaction and result in burnout syndrome. Conway et al. indicated that lower technical titles were correlated with relatively high turnover, whereas those with higher technical titles stayed in jobs for longer [42]. Spending longer in a position corresponds with good work experiences and more resources and support from supervisors, which may reduce burnout syndrome. Similarly, those with administrative duties have more opportunities for promotions and greater social resources, and thus have less risk of suffering from burnout syndrome.

In models 1 and 2 of our general linear model analysis, gender and technical title were not associated with burnout syndrome; rather, these two factors became significant in the combined analysis with self-reported social support and job satisfaction in Model 3. Therefore, the relationships between burnout syndrome and gender and technical title may be influenced by self-reported social support and job satisfaction.

Three strengthens of this study should be mentioned. First, a self-administered questionnaire was used, and subjects were unaware of the research hypothesis. Therefore, information bias could be avoided. Second, Sichuan Province has a population of about 80 million, accounting for 41.67% of the population of southwest China, and is home to 55.18% of southwest China’s medical workers [43]. Thus, our sample is likely representative of southwest China. Third, our study was the first to investigate the association of work-family conflict, self-reported social support, and job satisfaction with burnout syndrome in medical workers of southwest China.

Two limitations should be considered. First, the questionnaire used is not an international questionnaire, but was developed to fit conditions in China. However, the questionnaire has shown good reliability and validity [36]. Second, this was a cross-sectional study, meaning it had low power to detect causal effects. Therefore, perspective studies are needed to confirm our findings.

## Conclusions

In conclusion, the present study suggests that work-family conflict and self-reported social support slightly affect the level of burnout syndrome, while job satisfaction greatly influences burnout among medical workers of southwest China. Moreover, females and those with lower technical titles and without administrative duties are more vulnerable to burnout syndrome than their counterparts. Our study provides evidence that should be considered by health management institutions as they establish policies to prevent burnout syndrome in medical workers of southwest China.

## Supporting information

S1 File STROBE checklist. (DOC)

S1 Table The survey questionnaire in Chinese. (PDF)

S2 Table The survey questionnaire in English. (PDF)
