# Supplementary material for: Relationship of work-family conflict, self-reported social support and job satisfaction to burnout syndrome among medical workers in southwest China: A cross-sectional study
Source: PLoS One. 2017 Feb 16;12(2):e0171679. doi: 10.1371/journal.pone.0171679 (PMC5312880; doi:10.1371/journal.pone.0171679)
Supplement: S1 Table — (PDF) [file pone.0171679.s002.pdf]

表　　号：国卫调 3 表
制定机关：国家卫生计生委
批准机关：国家统计局
批准文号：国统制[2013]65 号
有效期至：2013 年 12 月

医务人员调查表

单位地址：

________省（市、区）________县（市、区）____________街道(乡镇)

机构名称：______________________________

尊敬的各位医务人员：

您好！医务人员调查是第五次国家卫生服务调查的重要组成部分，其目的是了解医务人员的执业状况、工作状态等情况，为制定国家的医疗卫生政策以及改善管理提供依据。

研究结果的可信度取决于您对问题的认真和客观回答，请您填写此问卷时，细心阅读各项问题，真实地表达您的感受。本调查严格按照《中华人民共和国统计法》要求进行，不记姓名，答案没有对错之分，我们将对调查资料严格保密，请不要有任何顾虑。

谢谢您的支持与合作。

表1、个人基本情况（在右侧空栏处填写数字）

序号	问题及选项	回答
1	性别：(1)男 (2)女	
2	年龄（岁）	
3	婚姻状况：(1)未婚 (2)已婚 (3)离婚 (4)丧偶 (5)其它	
4	最高学历：<br>(1)博士研究生 (2)硕士研究生 (3)大学本科 (4)大专<br>(5)中专/中技 (6)技工学校 (7)高中 (8)初中及以下	
5	专业技术职称：<br>(1)正高 (2)副高 (3)中级 (4)师（初）级 (5)士级 (6)无职称	
6	执业资格：<br>(1)执业医师 (2)执业助理医师 (3)中医执业医师<br>(4)中医执业助理医师 (5)注册护士 (6)其它	
7	目前主要从事专业类别：(1)临床医疗 (2)护理 (3)公共卫生	
8	工作年数（年）	
9	您现在工作的机构类型：(1)医院 (2)社区卫生服务中心 (3)乡镇卫生院	
10	在现机构工作年数（年）	
11	是否为本机构正式编制人员：(1)是 (2)否 (3)不知道	
12	所在科室是：<br>(1)内科 (2)外科 (3)妇产科/儿科<br>(4)中医科 (5)预防保健（公共卫生） (6)其他临床科室	
13	行政业务管理职务：<br>(1)院长、中心主任 (2)副院长、中心副主任 (3)科室主任<br>(4)科室副主任 (5)护士长 (6)无管理职务	
14	您平均每周工作多少小时？	
15	您平均每月在单位值夜班（不包括在家听班）的次数？	
16	去年，您总的年收入是多少（元）？（含工资、奖金及各种补贴等）	

表 3、工作感受（1）（在最符合您状况的那个数字上面划圈）

以下问题请根据您自身的感受在对应的符合程度上打圈

1. 非常不符合　2. 比较不符合　3. 有点不符合　4. 有点符合　5. 比较符合　6. 非常符合

1	工作对我来说是一个学习和成长的过程	1	2	3	4	5	6
2	通过工作，我的知识和技能在逐步提升	1	2	3	4	5	6
3	在工作中我可以尝试一些新事物，积极挖掘自身潜能	1	2	3	4	5	6
4	现在的工作对我的个人成长没有任何帮助	1	2	3	4	5	6
5	我对医院里的同事非常满意	1	2	3	4	5	6
6	我对我所从事的工作本身非常满意	1	2	3	4	5	6
7	我对医院内的提升机会非常满意	1	2	3	4	5	6
8	我对我从医院得到的报酬非常满意	1	2	3	4	5	6
9	我对医院内的环境条件非常满意	1	2	3	4	5	6
10	我对医院内的设备条件非常满意	1	2	3	4	5	6
11	总体来说，我对我目前的工作非常满意	1	2	3	4	5	6
12	我对我的直接上级非常满意	1	2	3	4	5	6
13	总的来说，我感觉工作压力很大	1	2	3	4	5	6
14	总的来说，我感到工作的紧张程度很高	1	2	3	4	5	6
15	我因为工作而难以入睡	1	2	3	4	5	6
16	我因为工作而紧张不安	1	2	3	4	5	6
17	我经常想离开这家医院	1	2	3	4	5	6
18	我经常想离开我现在所从事的行业	1	2	3	4	5	6
19	最近，我经常想换一下工作	1	2	3	4	5	6
20	明年我很有可能会找一份新工作	1	2	3	4	5	6

表 4、工作感受（2）（在最符合您状况的那个数字上面划圈）

以下问题请根据您在工作中的真实感受，判断您在工作中是否有过如下感觉。如果您从来没有这种感受，请选择数字“0”。如果您曾有过这样的感受，请在最能反映您感受的频繁程度的相应数字上画圈。

0. 从不（从来没有）

1. 极少（一年几次或更少） **2. 偶尔（一个月一次或更少）** **3. 有时（一个月几次）**

4. 经常（每周一次） **5. 频繁（一周几次）** **6. 总是（每天）**

1	在工作中，我感到自己迸发出能量	0	1	2	3	4	5	6
2	我觉得我所从事的工作目的明确，且很有意义	0	1	2	3	4	5	6
3	当我工作时，时间总是过得飞快	0	1	2	3	4	5	6
4	工作时，我感到自己强大而且充满活力	0	1	2	3	4	5	6
5	我对工作充满热情	0	1	2	3	4	5	6
6	工作时我会忘记周围的一切	0	1	2	3	4	5	6
7	工作激发了我的灵感	0	1	2	3	4	5	6
8	早上一起床，我就想要去工作	0	1	2	3	4	5	6
9	当我紧张工作时，我会感到快乐	0	1	2	3	4	5	6
10	我为我所从事的工作感到自豪	0	1	2	3	4	5	6
11	我沉浸于自己的工作当中	0	1	2	3	4	5	6
12	我可以一次连续工作很长时间	0	1	2	3	4	5	6
13	对我来说，我的工作具有挑战性	0	1	2	3	4	5	6
14	我在工作时会达到忘我的境界	0	1	2	3	4	5	6
15	工作时，即使感到精神疲劳，我也能很快地恢复	0	1	2	3	4	5	6
16	我感觉到自己离不开这份工作	0	1	2	3	4	5	6
17	即使工作进展不顺利，我也总能够锲而不舍	0	1	2	3	4	5	6

表 5、工作与家庭（在最符合您状况的那个数字上面划圈）

	1. 非常不同意　2. 比较不同意　3. 不能确定　4. 比较同意　5. 非常同意					
1	我的工作阻碍我参加家庭活动比我想象的多	1	2	3	4	5
2	我花在工作上的时间必然影响我参与家庭活动的时间	1	2	3	4	5
3	由于我必须把时间花在工作上，我常错过家庭活动	1	2	3	4	5
4	解决工作中问题的方法，不能帮助我解决我的家庭问题	1	2	3	4	5
5	在用解决工作中问题的方法解决家庭问题时，总是适得其反	1	2	3	4	5
6	我在工作上令我有出色表现的方法，不能帮我成为一个好的父母或配偶	1	2	3	4	5
7	当我下班后，我经常疲累得提不起劲去参与家中的活动	1	2	3	4	5
8	下班后我经常感到心力交瘁，妨碍我对我家中做出贡献	1	2	3	4	5
9	由于工作中种种压力，有时就算回到家中也不愿多干自己喜欢的事情	1	2	3	4	5
