# Supplementary material for: Relationship of work-family conflict, self-reported social support and job satisfaction to burnout syndrome among medical workers in southwest China: A cross-sectional study
Source: PLoS One. 2017 Feb 16;12(2):e0171679. doi: 10.1371/journal.pone.0171679 (PMC5312880; doi:10.1371/journal.pone.0171679)
Supplement: S2 Table — (PDF) [file pone.0171679.s003.pdf]

No. of questionnaire: 3th of NHSS
Design by: National Health and Family Planning Commission of the People's Republic of China
Approval authority: National Bureau of Statistics
No. of license: No.65 of Guotongzhi[2013]
Expiration Date: December 2013

Medical staff questionnaire

Hospital address:
Hospital name:

Dear medical staff,

Medical staff survey is an important part of the Fifth National Health Service Survey, which aims to know the practice status and work status of medical staff, and provides the evidence for the establishment of national health policy and the improvement of management.

The credibility of the results depends on your serious and objective answers to the questions. Please read the questions carefully and express your real feelings when you fill out this questionnaire. This survey is conducted in strict accordance with the requirements of *The statistics law of the People's Republic of China*. The names of the respondents are not recorded. The answers are not divided into right and wrong. We will keep the information strictly confidential. Please do not have any concerns.

Thanks for your support and cooperation.

Table 1 Basic personal information (Fill in the blank at the right side with figures)

No.	Questions & Options	Answers
1	Sex: (1)Male (2)Female	
2	Age(years)	
3	Marital status: (1)unmarried (2)married (3)divorced (4) widowed (5)others	
4	Highest educational level: (1)doctor (2)master (3)bachelor (4) junior college (5)technical secondary school (6)skilled worker's school (7)high school (8)junior high school and below	
5	Professional title: (1)senior title (2)vice-senior title (3)middle title (4)primary title (5)assistant title (6)no title	
6	Practice qualification: (1) practicing physician (2) assistant practicing physician (3)Chinese medicine practitioner (4) assistant practicing doctors of traditional Chinese medicine (5)registered nurse (6)others	
7	Main professional category: (1)clinical medicine (2)nursing (3)public health	
8	Years of work	
9	Type of work organization: (1)hospital (2)community health service (3) township hospital	
10	Years of work in this organization	
11	Whether you are the formal staff in this organization: (1)yes (2)no (3)don't know	
12	Department: (1)medical department (2) surgical department (3) obstetrics and gynecology department/department of pediatrics (4) traditional Chinese medicine department (5)prevention and health department (6)other clinical departments	
13	Administrative position: (1) director of the hospital/center director (2)deputy director of the hospital/center deputy director (3)director of department (4)deputy director of department (5)head nurse (6)no administrative position	
14	How many hours per week do you work on average?	
15	The days you are on night duty in the organization every month on average.	
16	What was your annual income in the last year(Yuan)? (including salary, bonus and subsidy)	

Table 3 Work feelings (1)

Please circle the appropriate option according to your own feelings 1.definitely false 2.mostly false 3.a little false 4.a little true 5.mostly true 6.definitely true							
1	Work is a learning and growing process for me .	1	2	3	4	5	6
2	My knowledge and skills are gradually improving through work.	1	2	3	4	5	6
3	In the work I can try some new things, and find out my own potential capacity.	1	2	3	4	5	6
4	My current job does not help my personal growth.	1	2	3	4	5	6
5	I am very satisfied with my colleagues in the hospital.	1	2	3	4	5	6
6	I am very satisfied with my jobs.	1	2	3	4	5	6
7	I am very satisfied with the promotion in the hospital.	1	2	3	4	5	6
8	I am very satisfied with my salary in the hospital.	1	2	3	4	5	6
9	I am very satisfied with the hospital environment.	1	2	3	4	5	6
10	I am very satisfied with the hospital equipment.	1	2	3	4	5	6
11	In general, I am very satisfied with my current job.	1	2	3	4	5	6
12	I am very satisfied with my immediate superior.	1	2	3	4	5	6
13	In general, I have a lot of work pressure.	1	2	3	4	5	6
14	In general, I feel the work tension is high.	1	2	3	4	5	6
15	I can not sleep easily because of my work.	1	2	3	4	5	6
16	I feel nervous because of my work.	1	2	3	4	5	6
17	I always want to leave this hospital.	1	2	3	4	5	6
18	I always do not want to engage in this industry.	1	2	3	4	5	6
19	Recently, I always want to change my job.	1	2	3	4	5	6
20	I will probably find a new job in the next year.	1	2	3	4	5	6

Table 4 Work feelings (2)

Please determine whether you have the following work feelings, basing on your real feelings. If you never have such feelings, please choose "0". If you have such feelings, please circle the appropriate figure.<br>0.never(none of the time) 1.seldom(several times a year or less) 2.occassionaly(once a month or less) 3.sometimes(several times a month) 4. often (once a week) 5.frequently(several times a week) 6.alwyas(every day)								
1	I am full of energy in the work.	0	1	2	3	4	5	6
2	I think my job is motivated and meaningful.	0	1	2	3	4	5	6
3	When I work, time flies.	0	1	2	3	4	5	6
4	When I work, I feel strong and energetic.	0	1	2	3	4	5	6
5	I am passionate about my work.	0	1	2	3	4	5	6
6	I will forget everything around me when I work.	0	1	2	3	4	5	6
7	Work inspires me.	0	1	2	3	4	5	6
8	Once I get up in the morning, I want to go to work.	0	1	2	3	4	5	6
9	I will feel happy when I work intensively.	0	1	2	3	4	5	6
10	I feel proud of my job.	0	1	2	3	4	5	6
11	I am immersed in my work.	0	1	2	3	4	5	6
12	I can work continuously for a long time.	0	1	2	3	4	5	6
13	My job is challenging.	0	1	2	3	4	5	6
14	I work selflessly.	0	1	2	3	4	5	6
15	When I work, I can recover quickly even if I am tired.	0	1	2	3	4	5	6
16	I feel I can not leave this job.	0	1	2	3	4	5	6
17	I can always persevere even if the work does not go well.	0	1	2	3	4	5	6

Table 5 Work & Family (Please circle the appropriate option according to your condition)

1.strongly disagree 2.partly disagree 3.not clear 4.partly agree 5.strongly agree						
1	My work prevents me from participating in family activities more than I think.	1	2	3	4	5
2	The time I spend at work inevitably affects my time at home.	1	2	3	4	5
3	I often miss family activities because I have to spend time on work.	1	2	3	4	5
4	I can not solve my family problem using the way which I use to solve my work problem.	1	2	3	4	5
5	When I solve my family problem using the way which I use to solve my work problem, the result will always turn out to be just the opposite of my wish.	1	2	3	4	5
6	The way I do my job perfectly does not help me to be a good parent or spouse.	1	2	3	4	5
7	I am always too tired to participate in family activities after work.	1	2	3	4	5
8	I always feel exhausted after work, which prevents me from contributing to the home.	1	2	3	4	5
9	Sometimes, I don’t want to do what I like when I return home because of pressures in work.	1	2	3	4	5

Table 7 Working environment (Fill in the blank at the right side with figures)

No.	Questions & Options	Answers
1	In your opinion, do your patients respect you? (1)very respectful (2)partly respectful (3)moderate (4)partly disrespectful (5)very respectful	
2	In your opinion, does the society respect your vocation? (1)very respectful (2)partly respectful (3)moderate (4)partly disrespectful (5)very respectful	
3	In your opinion, which point in the scale is the current social status of medical staff status?(0 is the lowest, 100 is the highest)	
4	In your opinion, the degree of the patients' trust in your service is: (1)very high (2)a bit high (3)moderate (4)a bit low (5) very low	
5	In your opinion, the current doctor-patient relationship is: (1)very good (2)a bit good (3)moderate (4)a bit bad (5) very bad	
6	In the recent 6 months, you have received the patients': (1)insult (2)physical violence (3)both (4)neither	
7	In most cases, on your service, patients will feel: (1)very satisfied (2)partly satisfied (3)noncommittal (4)partly dissatisfied (5)very satisfied	
